# Three-Dimensional Culture of Epithelial Cells on Electrospun Nanofibrous Scaffolds

**DOI:** 10.3390/ijms262110500

**Published:** 2025-10-29

**Authors:** In-Jeong Lee, Jong-Young Kwak

**Affiliations:** 1 3D Immune System Imaging Core Center, School of Medicine, Ajou University, Suwon 16499, Republic of Korea; injeong@ajou.ac.kr; 2 Department of Pharmacology, School of Medicine, Ajou University, Suwon 16499, Republic of Korea

**Keywords:** epithelium, epithelial cell, nanofiber, scaffold, three-dimensional culture

## Abstract

Epithelial tissues form protective barriers throughout the body, covering external surfaces and lining internal cavities. Nanofibrous scaffolds have emerged as leading platforms in tissue engineering because of their ability to mimic the nanoscale fibrillar architecture of the native extracellular matrix. Thus, they support the optimal microstructure and cellular functions that facilitate the generation of epithelial tissues. This review focuses on the pivotal role of nanofibrous scaffolds in the development of physiologically relevant three-dimensional (3D) culture systems for various types of epithelial cells. Nanofiber proper ties, including diameter, alignment, and surface chemistry, can be tailored to modulate epithelial cell attachment and growth on scaffolds. Fabrication techniques and optimized scaffold properties for culturing epithelial cells from various epithelial tissues on nanofibrous scaffolds have been examined. The key 3D culture methodologies and coculture systems that incorporate fibroblasts, endothelial cells, and immune cells, which are essential for achieving functional differentiation into an epithelium, are elucidated. Finally, the current challenges in this field and potential future directions, including the integration of scaffolds into organ-on-a-chip systems, development of “smart” bioactive materials, and pursuit of personalized medicine through patient-derived cells, are discussed.

## 1. Introduction

**Figure 1:**
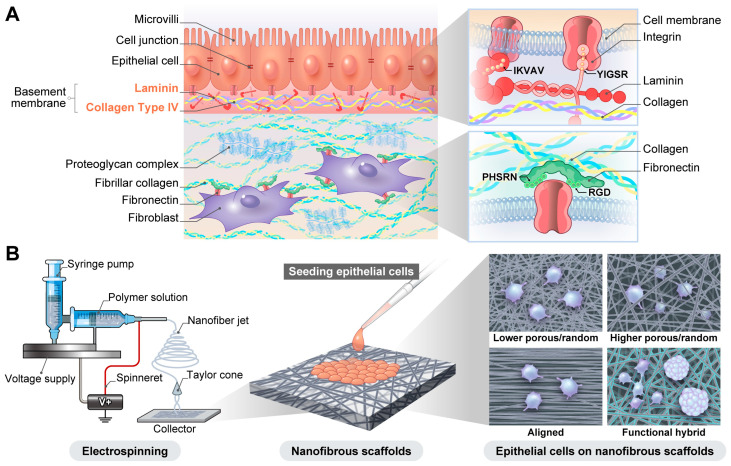
Schematic representation of epithelial cell 3D culture on nanofibrous scaffolds for developing in vivo-mimicking epithelial tissues. (**A**) Attachment of epithelial cells to the basement membrane in the epithelial tissue. Integrins in the surface of epithelial cells bind to specific molecules within the basement membrane, such as laminin. (**B**) Electrospinning of nanofibers and culture of the epithelial cells on the nanofibrous scaffold. The attachment and growth of epithelial cells on the nanofibrous scaffolds are influenced by the scaffold’s polymer type, alignment, and porosity.

Epithelial cells attach to the basement membrane (BM), a specialized extracellular matrix ECM layer that provides adhesion sites via surface receptors [1]. Additionally, ECM fibers such as collagen regulate cell migration and adhesion through variations in fiber density and diameter [2]. The primary class of surface receptors that interact with the ECM is integrins, a family of heterodimeric proteins that bind to a range of ECM constituents, including fibronectin, vitronectin, laminin, and collagen (Figure 1A) [3]. Integrin receptors on epithelial cell surfaces bind to laminins, a significant component of the BM, to facilitate cell adhesion and signaling [4]. Thus, laminin connects the cells to collagen fibers in the ECM [5].

Three-dimensional (3D) cell culture is a promising alternative to traditional 2D cell cultures and animal models. It offers a more cost-effective and ethically sound approach for various research and drug development applications [6,7]. For example, in cancer research, 3D cultures provide an environment similar to the tumor microenvironment (TME) in vivo, whose replication using 2D culture methods is challenging. This enables a more accurate study of the complex structures of tumors, intercellular interactions, and drug reactions [8,9]. When cultured on flat, rigid plastic surfaces, epithelial cells adopt an unnatural morphology, lose their distinct polarity, and fail to establish the complex cell–cell and cell–matrix interactions that govern tissue function in vivo. Notably, primary bronchial epithelial cells isolated from the airways dedifferentiate when propagated as monolayers in submerged culture [10].

Various biomimetic BM scaffolds, including porous polymeric membranes, hydrogels, and electrospun membranes, have been developed [11]. Among the various scaffold architectures developed for 3D culture models, nanofiber-based scaffolds have emerged as promising platforms for tissue engineering. Nanofibrous scaffolds provide an ideal environment for cell culture, promoting cell adhesion, growth, and proliferation owing to their architectural resemblance to the natural ECM, offering a high surface area and interconnected porosity (Figure 1B). Conventional approaches for differentiated epithelial cell culture are based on Trans-well systems [12], where cells form polarized monolayers on porous membranes, creating independent apical and basolateral compartments, and thus mimicking some basic properties of in vivo epithelial tissues. Notably, 3D bioprinting is a relatively recent and versatile manufacturing technique that enables the layer-by-layer construction of tissues and microtissues using bioinks derived from cell-laden materials [13,14]. Microfabrication techniques offer new alternatives for achieving a more accurate representation of complex BMs in native epithelial tissues [11,15]. Several lab-on-a-chip devices capable of representing most epithelial barriers in the human body have been designed and maintained in a functional state for several weeks [16]. Because of their ability to create micro-physiological similarities, electrospun nanofibrous scaffolds have become invaluable for developing in vitro disease models and high-throughput drug screening. Interestingly, Scuto et al. report that micro- and nanoplastics can potentially induce tumor initiation and progression, particularly through enhancing cell migration and possibly fueling metastasis [17]. Thus, integrating advanced technologies, such as organ-on-a-chip platforms, nanofibrous membranes, and machine learning, along with identifying key molecular targets, lays the foundation for developing precision and personalized medicine strategies aimed at lowering the risk of environmentally induced carcinogenesis [17]. For example, nanofibrous scaffolds can be fabricated to detect micro- and nano-plastics pollution [18,19].

This review focuses on the critical interactions between epithelial cells and nanofibrous scaffolds, emphasizing the evolution of scaffold design from providing passive, biologically inert support to delivering active, biologically instructive signals that direct the formation of complex tissues. In addition, specific applications across various epithelial systems are examined, and the persistent challenges hindering clinical translation are critically evaluated. Thus, the results demonstrate that by providing a suitable environment for cell attachment and growth, nanofibrous scaffolds can be utilized for regenerating epithelial tissues.

## 2. Nanofibrous Scaffold Fabrication by Electrospinning

Various fabrication methods are used for nanofibrous scaffolds, each with distinct advantages. While electrospinning dominates, other techniques exist, including template synthesis, self-assembly, phase separation, melt blowing, and drawing, each with its own advantages and limitations compared to electrospinning [20,21]. These alternatives offer specific applications, such as producing highly ordered structures, large-scale production, and specialized materials, which may not be suitable for traditional electrospinning. Electrospinning is the most widely used technique for fabricating continuous nanofibers for tissue scaffolds [22,23]. The process involves ejecting a polymer solution through a nozzle under a high-voltage electrostatic field. Electrospinning techniques are prevalent owing to their simplicity, low cost, scalability, and ability to produce fibers with high surface area and porosity [24]; types include needleless, coaxial, tri-axial, and multi-needle electrospinning, enabling diverse fiber structures [25]. A nanofibrous scaffold can be tailored by controlling its physical, mechanical, and biological properties. By modulating parameters such as voltage, solution concentration, and flow rate, it is possible to control fiber diameter, morphology, and orientation. The diameter of nanofibers influences the surface topography for cell interaction. Interconnected porosity is essential for cell infiltration. Once adhered, epithelial cells must proliferate to cover the scaffold surface. The porous architecture supports this expansion by providing space and ensuring nutrient availability. Nanofiber alignment can direct cell behavior; random fibers promote uniform cell sheet formation, while aligned fibers guide directional cell migration. Kidney epithelial cells followed polycaprolactone (PCL) fiber patterns on aligned and random fiber membranes, changing their morphology [26]. The nanoscale topography provides sites for focal adhesions anchoring cells to the substrate. This interaction triggers integrin-mediated signaling cascades regulating cell behaviors like spreading and proliferation. The mechanical environment influences cell behavior, with flexible scaffolds needed for skin and stiffer ones for corneal regeneration. Understanding how nanofiber scaffolds influence epithelial cell behavior is necessary to optimize epithelial tissue designs. For many epithelial tissues, a simple monolayer is insufficient; they require a complex structure. Creating functional and multilayered tissue requires both physical support and biochemical instruction from the scaffold.

## 3. Polymer Scaffolds for Epithelial Cell Adhesion and Growth

The choice of polymer for nanofiber fabrication is fundamental to scaffold design, with natural, synthetic, and hybrid materials each offering a distinct profile of advantages and disadvantages (Table 1). Materials are broadly classified into natural and synthetic polymers, with hybrid composites emerging as a powerful third category.

### 3.1. Natural Polymers

Polymers such as collagen, gelatin, silk fibroin (SF), chitosan, and hyaluronic acid resemble the components of natural ECM and are frequently used to fabricate nanofibers for biomedical applications [27,28]. They inherently possess good biocompatibility, biodegradability, and bioactivity, actively promoting cell adhesion, proliferation, and epithelialization [28]. The reaction of cells to natural polymers varies depending on the material. Gelatin contains arginine–glycine–aspartate (RGD) sequences that enhance cell attachment and are widely used owing to their bio-affinity [29]. However, natural polymers exhibit poor mechanical strength, immunogenic responses, and significant batch-to-batch variability, which restrict their applicability in nanofiber production [30].

### 3.2. Synthetic Polymers

Synthetic polymers, such as PCL, poly(lactic acid) (PLA), poly(L-lactic acid) (PLLA), and poly(lactic-co-glycolic acid) (PLGA), are widely used in nanofiber synthesis and tissue engineering owing to their tunable mechanical properties, controllable degradation rates, and high purity and batch-to-batch consistency [31]. While synthetic polymers are often selected for their inertness within biological systems, their degradation can produce acidic byproducts, some of which can induce local inflammation [32,33]. Thus, synthetic polymers are chosen for tunable mechanics and reproducibility, but their biodegradation and surface chemistry require careful mitigation, including blending, coatings, and buffering, to avoid adverse effects [32]. Moreover, innovative applications of synthetic scaffold-based drug delivery in 3D systems are emerging for the controlled release of therapeutic agents in disease models [29]. Synthetic scaffolds are capable of sequential drug release, transitioning to cell-friendly surfaces, and enabling combinatorial therapy. In 3D culture conditions, we also need to consider conventional therapeutic drugs with nanocarrier delivery systems as hormetic drugs, as they can control cell proliferation in a dose-dependent manner [34,35]. Therefore, the concept of hormesis (a dose-response mechanism) should be applied to drug-delivery systems in 3D platforms for personalized disease models.

### 3.3. Hybrid Polymers

Synthetic polymers, such as PLA and PCL, are characterized by tunable mechanical strength and finer fiber diameters. However, they often lack the biological cues necessary for optimal cell interaction. Thus, combining natural and synthetic polymers in composite or blended scaffolds has emerged as a powerful strategy for synthesizing materials with synergistic properties. Various approaches have been employed to modify synthetic polymer scaffolds with specific bioactive properties; for instance, in surface immobilization, proteins and peptides are directly attached to nanofiber surfaces to enhance cell-specific interactions. Covalent bonding is generally preferred over simple physical adsorption as it creates a more stable and durable linkage, preventing premature detachment of biomolecules. For example, natural and synthetic polymers, such as PCL/chitosan, PCL/gelatin, and collagen-coated polyethylene terephthalate (PET), are combined to form a superior composite material that exhibits optimal physicochemical properties, mechanical stability, and bioactivity while mitigating the drawbacks of single materials [36,37]. However, balancing the properties of each component is complex and requires careful optimization. Full-length proteins, such as collagen, laminin, and fibronectin, provide a rich array of signaling domains, whereas short and specific peptide sequences (such as RGD) can be used to target particular integrin receptors and promote cell adhesion. Hybrid composites can be expanded to include biologically modified scaffolds. Recently, mesenchymal stem cell-conditioned media doped onto TiO_2_ nanoparticles demonstrated that stem cell secretome factors can synergize with nanomaterials, improving mechanical stability, bioactivity, and anti-inflammatory potential in engineered scaffolds [38].

### 3.4. Laminin and Laminin-Derived Peptide-Blended Nanofibers for Epithelial Cell Culture

Laminins, which are significant thin-layer components of the BM, separate epithelial cells from the underlying tissue and act as ligands for integrins on the epithelial cell surface, facilitating cell attachment to the BM [39,40]. Laminins also play a crucial role in establishing and maintaining the apicobasal polarity of epithelial cells [40]. Compared to traditional, unmodified nanofiber scaffolds, nanofibers functionalized with laminins may provide a more natural environment for epithelial cells. Submicron or nanoscale fibers of laminin proteins are generated by electrospinning a laminin solution, which mimics the morphological properties and bioactivity of BMs [41]. Baskapan and Callanan successfully electrospun laminins in PCL scaffolds [42]. The PCL nanofibrous scaffolds are enriched with laminins either by direct blending with polymer solutions or as an emulsion with a surfactant. Renal epithelial cells on a laminin-blended PCL nanofibrous scaffold are metabolically active and show expression of essential genes in the renal cells. Poly(vinyl alcohol) (PVA) nanofibrous scaffolds, containing integrin-binding peptides of laminins, promote peptide-specific adhesion and growth of epithelial cells. Tran et al. fabricated PVA nanofibers containing cell-adhesive peptides, such as Tyr-Ile-Gly-Ser-Arg (YIGSR) and Ile-Lys-Val-Ala-Val (IKVAV), derived from laminins for culturing bronchial epithelial cells on nanofibrous scaffolds [43]. These bronchial epithelial cells, cultured on the laminin-derived peptide-retained nanofibrous scaffold, formed layers instead of cell aggregates and spheroids. Their growth patterns were similar to those of the cells cultured on a laminin-coated nanofibrous scaffold [43]. PLGA nanofibrous scaffolds provide signals to salivary gland epithelial cells, prompting them to proliferate and initiate apicobasal polarity in cultures [44]. Laminin-111-functionalized PLGA nanofibrous scaffolds promote the apicobasal polarity of epithelial cells by stimulating the apical localization of tight proteins, including Zonula Occludens (ZO)-1 [44]. These results indicate that functionalized nanofibers with laminins provide the specific biochemical signals necessary to mimic the native BM.

## 4. Tissue-Specific Application of Nanofibrous Scaffolds

### 4.1. Bronchial and Lung Epithelial Cell Culture on Nanofibrous Scaffolds

The human lung is a masterpiece of biological engineering with a complex hierarchical architecture for efficient gas exchange. This process occurs at the alveolar-capillary interface, a barrier comprising alveolar epithelial cells, capillary endothelial cells, and their fused BM. The upper airways, lined with pseudostratified mucociliary epithelium, provide critical defense through mucociliary clearance. Commercially available 3D culture inserts with porous polymer membranes enable the establishment of an air–liquid interface (ALI), necessary for mucociliary differentiation [45]. Trans-well-based ALI systems remain a widely used, physiologically relevant platform when appropriately designed [46]. While some flat polymer membranes (rigid, non-fibrillar) do poorly recapitulate basement-membrane nanoscale topography, many modern porous membranes and modified inserts (e.g., ECM-coated Trans-wells, nano/micro-patterned membranes, or electrospun nanofiber membranes adapted to ALI culture) can support physiologically relevant mucociliary differentiation, barrier function, and host–pathogen responses when properly prepared. Many studies utilize ALI conditions, but protocols differ across the same cell line [45]. Thus, tuning the biochemical and mechanical properties of nanofiber scaffolds through polymer selection and fabrication parameters allows the creation of in vitro lung models with unprecedented physiological relevance.

#### 4.1.1. Inadequacy of Conventional Models and Emergence of a 3D Culture of Bronchial and Lung Epithelial Cells

The human airway epithelium is a sophisticated barrier against pathogens, pollutants, and allergens. Its integrity and complex functions, including mucociliary clearance, innate immune signaling, and tissue repair, are central to respiratory health. Researchers have sought to model this system in vitro to study disease mechanisms and develop therapies. However, traditional models have consistently lacked the full complexity of human bronchial and lung pathophysiology. Notably, 2D culture models of bronchial and lung epithelial cells cannot achieve complete mucociliary differentiation, making them unsuitable for studying barrier function or host-pathogen dynamics realistically. While animal models offer systemic complexity, they are limited by significant anatomical, physiological, and immunological differences from human lungs. These disparities often lead to poor translation of preclinical findings, contributing to high failure rates in clinical trials and increased costs. By replicating the physical and topographical cues of the natural cellular microenvironment, nanofibrous scaffolds provide an opportunity to construct physiologically relevant 3D lung models, that can revolutionize respiratory research. The development of airway/alveolar models, including nanofiber membranes integrated into ALI systems, indicates that the application extends beyond primarily cancer research [47].

#### 4.1.2. Cell Sources: Building Blocks of the Model

The choice of lung epithelial cell type is a critical determinant of the physiological relevance and reproducibility of the model. The two primary cell types, each with distinct advantages and disadvantages, are primary cells and immortalized cell lines (Table 2). Primary human cells, such as normal human bronchial epithelial (NHBE) cells and small airway epithelial cells (HSAEC), offer superior physiological fidelity and can differentiate into a complex, pseudostratified mucociliary epithelium at an ALI, mimicking the native airway. In comparison, A549 (human adenocarcinomic alveolar basal epithelial cells), H441 (human lung papillary adenocarcinoma), and Calu-3 (human lung adenocarcinoma) cells are robust and reproducible, making them suitable for initial studies; however, their cancerous origin can limit their physiological fidelity.

#### 4.1.3. Nanofibrous Scaffolds for 3D Cultures of Bronchial and Lung Epithelial Cells

Once seeded onto the nanofibrous scaffold, lung epithelial cells are cultured to promote differentiation using the ALI method in static or dynamic systems [48,49]. This technique induces mucociliary differentiation of airway epithelial cells, typically performed in Trans-well^®^ inserts using ALI medium, where the nanofibrous scaffold acts as a porous membrane. NHBE cells differentiate on the PCL nanofiber layer during ALI culture [50,51]. Goblet and ciliated cells were observed 14 and 21 days after ALI initiation [51]. Choi et al. demonstrated that NHBE cells form a normal pseudostratified epithelium with ciliated, goblet, and basal cells on a thin-layer (six-layer) PCL mesh [50]. However, cells on thick-layer (80-layer) PCL mesh differentiate into hyperplastic goblet cells via epithelial-mesenchymal transition (EMT) and oxidative stress. This shows that nanofibrous scaffold thickness affects human bronchial epithelial cell differentiation. Radiom et al. used gelatin nanofibers with hexagonal geometry, imitating alveolar air sacs to replicate the alveolar air-tissue interface [52]. A549 cells adhered well to the gelatin nanofibers, reaching 90% confluency after several days of culture. Fibroblasts and epithelial cells respond differently to substrate properties influencing nanoparticle uptake [53]. However, fibroblasts (MRC-5 cells) were higher on aligned and nonaligned polyurethane fiber scaffolds than on tissue culture plastic [54]. In contrast, epithelial cells (A549 cells) showed no increase.

When bronchial epithelial MLE-12 cells were cultured on PVA nanofibrous scaffolds with diameters of 150–250 nm and micropores, they formed multiple layers instead of cell aggregates and spheroids, and their growth patterns were similar to those of cells in epithelial tissue [43,55]. The diameter and pore size of the nanofibrous PVA membrane make it an ideal substrate for supporting the adhesion and growth of cells that mimic epithelial cells on the BM in epithelial tissues. PVA/collagen and PVA/silk sericin nanofibers with different diameters induce A549 cells to undergo EMT, which depends on the diameters of the nanofibers [56,57]. The cells interacted with the nanofibrous topological surface. This EMT transition may be induced under specific nanofiber topological conditions, owing to the cancerous characteristics of A549 cells.

#### 4.1.4. Coculture Systems Formed Using Nanofibrous Scaffolds

The airway wall is a complex tissue containing multiple cell types that engage in constant crosstalk. The alveolar-capillary barrier comprises three components: alveolar epithelium, capillary endothelium, and BM [58]. Coculture models, where two or more cell types grow together, are essential for recapitulating this complexity. Cocultures of epithelial cells and fibroblasts are critical for modeling the airway. Lung fibroblasts, cultured beneath or within the scaffold matrix, provide paracrine signals (e.g., growth factors and ECM components) that promote epithelial cell growth, enhance barrier integrity (as measured by transepithelial electrical resistance, or TEER), and modulate repair processes. The PCL nanofibrous scaffold, with diameters from 400 to 1500 nm and micropores of 10–50 μm, provides similar spatial dimensionality to the interstitial matrix for fibroblast growth [59]. The PCL nanofibrous scaffold supports the development and spatial organization of fibroblasts. Indirectly cocultured NIH3T3 cells stabilized MLE-12 epithelial cell attachment to the nanofibrous scaffold throughout the culture period [55]. This may be due to fibroblast-secreted factors, including collagen, fibronectin, laminin, and growth factors [60]. It has been demonstrated that lung tumors-on-a-chip, utilizing A549 cells and a PLGA electrospinning nanofiber membrane as the substrate for the microfluidic chip and the cell 3D culture scaffold [61]. The morphology of cultured A549 cells was spherical or ellipsoidal, embedded in PLGA nanofiber membrane pores, indicating 3D rather than 2D culture. When A549, human fetal lung fibroblasts (HFL1), and human umbilical vein endothelial cells (HUVECs) were cocultured, A549 cells induced endothelial cell apoptosis, leading to tumor cell invasion. An alveolar-capillary barrier model was developed using a PCL nanofibrous scaffold coated with collagen type I mimicking BM, human microvascular endothelial cells (ISO-HAS-1), and human lung adenocarcinoma cells (NCI H441) in bipolar coculture [62]. Triple-cultures of THP-1 macrophages, epithelial cells, and endothelial cells on collagen-coated PCL nanofibrous scaffold produced a thicker membrane than bipolar coculture, as epithelial cells organize into a multilayer with macrophages present.

#### 4.1.5. Applications of Nanofibrous Scaffolds in ALI Systems

To model the alveolar-capillary barrier (“air-blood barrier”), epithelial cells are cultured on one side of a nanofibrous scaffold, with endothelial cells on the opposite side. This arrangement is essential for studying gas exchange, particle translocation, and vascular responses to inflammation or inhaled toxicants. Culturing lung epithelial cells in Trans-wells allows culture at the ALI and enables cocultures with endothelial cells grown on the basal membrane side or culture plate bottom. The alveolar-capillary interface comprised human expandable lung epithelial cells from human pluripotent stem cells and HUVEC seeded on opposite sides of the PCL nanofibrous scaffold on UHELON mesh, which mimics the BM [63]. UHELON is a biocompatible mesh with a rectangular organization of polyamide fibers. Lung epithelial and HUVEC cells adhered to the PCL nanofibrous scaffold and proliferated during in vitro culture. Epithelial cells showed terminal differentiation, forming a compact epithelial layer and 3D structures with epithelial character, along with the presence of microvilli. This study showed that the PCL nanofibrous scaffold is suitable for creating a culture environment mimicking the alveolar-capillary interface of the human lung. Gabela-Zuniga et al. developed a ventilator-on-a-chip device using primary human alveolar epithelial cells and HMVEC-L coculture, a polyurethane nanofibrous scaffold, and microfluidics to simulate mechanical forces causing lung injury during ventilation [64]. Calu-3 bronchial epithelial cells were cultured on retinoic acid-loaded PCL-chitosan nanofibrous scaffold under ALI conditions for 14 days [65]. PCL-chitosan scaffolds supported Calu-3 cell viability, while retinoic acid release supported epithelial cell growth and increased mucociliary gene expression.

Epithelial cells can be cocultured with immune cells to study cellular crosstalk. For modeling infection and inflammation, immune cells like macrophages, neutrophils, or lymphocytes can be introduced via the apical surface or basal compartment. Neutrophils and macrophages migrated to *S. aureus*-infected MLE-12 bronchial epithelial cells in the PCL nanofibrous scaffold-based two-layer culture system [66]. The secretion of tumor necrosis factor (TNF)-α and interleukin (IL)-1α increased in lung epithelial cells cultured with *S. aureus* in 3D, but not in 2D culture, and *S. aureus*-infected epithelial cell detachment occurred only with migrating neutrophils from upper to lower PCL nanofibrous scaffold. These findings indicate an in vivo inflammation-mimicking response in 3D culture conditions. This enables investigation of host-pathogen interactions, immune cell recruitment, and inflammatory cytokine release in a human-relevant context.

### 4.2. 3D Culture of Retinal Epithelial Cells on Nanofibrous Scaffolds

Retinal pigment epithelium (RPE) cells form a monolayer in the outer retina, playing a crucial role in maintaining vision [67]. RPE dysfunction is a primary pathological change leading to retinal degenerative diseases, such as retinitis pigmentosa [68]. The RPE lies on Bruch’s membrane, a natural ECM compartment providing support for RPE cell adhesion, migration, and differentiation [69]. Bruch’s membrane loses its structural integrity and function over time [68]. In advanced retinal disease or macular surgery, the basal lamina layer of Bruch’s membrane may be damaged or absent [70]. Thus, artificial Bruch’s membrane-like scaffolds have been designed for RPE culture and implantation. Fibrous substrates are more similar in structure to Bruch’s membrane and enhance RPE cell function and viability, regardless of formulation [71]. In culturing RPE cells, a 3D environment should be provided to promote cell maturation, improve structural support, and facilitate cell survival and integration. The 3D structure of nanofibrous scaffolds can be designed to replicate the properties of Bruch’s membrane, the natural RPE basement membrane, aiding in the long-term integration of transplanted cells.

#### 4.2.1. Sources of RPE Cells

The development of RPE-scaffold constructs has utilized various cell sources, each with its own advantages and limitations [72]. The immortalized adult human RPE-19 (ARPE-19) cell line is a widely used tool for initial scaffold screening and proof-of-concept studies due to its robustness and ease of culture [73,74]. Primary human and porcine RPE cells provide a more biologically relevant model, closely recapitulating in vivo physiology. However, their use is constrained by limited donor availability, ethical considerations, and inter-donor variability [75,76,77]. Stem cell-derived RPE represents the most clinically relevant pathway for transplantation in retinal degenerative patients. Human embryonic stem cells can be differentiated into RPE under xeno-free conditions, providing a renewable and scalable source of clinical-grade cells [78]. However, transplanted differentiated RPE cells require a scaffold for residing.

#### 4.2.2. Nanofibrous Scaffolds for 3D Cultures of RPE Cells

Designing scaffolds that mimic the mechanical and topographical properties of native Brush’s membrane is crucial to support RPE cell polarization and function. This includes optimizing pore size, fiber diameter, and thickness. Thieltges et al. demonstrated that proliferating human primary RPE cells on a polyamide nanofibrous scaffold exhibited polygonal morphology in a colony-like distribution, suggesting a more epithelioid phenotype compared to cells on smooth surfaces, such as glass [79]. The surface topography and nanofiber architecture of the 3D nanofibrous scaffold had the most significant impact on RPE cells’ behavior, maturation, and organization [75]. No significant differences appeared between PLGA and collagen nanofibrous membranes regarding cell proliferation, size, organization, formation of microvilli, and tight junctions of cultured RPE cells [75]. This suggests that the structure of the biomaterial was more influential than the material itself in culturing epithelial cells. Human fetal RPE cells on PET and poly(l-lactide-co-ε-caprolactone) (PLCL) nanofibrous scaffolds with 200 nm fiber diameter exhibited the highest cell densities, resulting in adherent monolayers with deeper pigmentation and uniform hexagonal tight junctions [76]. Nanofibrous membranes showed favorable subretinal biocompatibility in the rabbit model. RPE cells cultured on polylactide nanofibrous scaffold showed better maturation and long-term survival compared to those on commercial polyester membrane [80]. The poly(l-lactide-*co*-dl-lactide) (PDLLA) nanofibrous scaffold showed higher porosity than the commercial membrane. It was more resistant to blockage by sub-RPE deposits owing to its open structure. However, the thin nanofiber scaffold’s disadvantage is lower stiffness compared to the commercial track-etched membrane. Nanofibrous scaffolds of hydrophobic polymer polyacrylonitrile (PAN) were treated with fluocinolone acetonide (FA) to increase hydrophilicity [81]. RPE cells survived and retained their characteristic morphology for up to 150 days using an FA-treated nanofibrous scaffold, exhibiting a morphological epithelial phenotype with biomarkers critical for retinal physiological characteristics. Recent research shows that scaffold mechanical stiffness is not merely passive, but an active signaling cue. In a direct comparative analysis, Surrao et al. demonstrated that RPE cells cultured on stiffer PLLA scaffolds exhibited significantly higher TEER values and greater phagocytic capacity compared to cells cultured on softer materials, such as PCL and PLGA [82]. This phenomenon, known as mechano-transduction, indicates that RPE cells can sense and respond to substrate mechanics, which in turn influences their maturation into a fully functional, barrier-forming epithelium. Nanofibrous scaffolds composed of PCL and PCL combined with collagen were identified as suitable for the long-term cultivation of RPE cells isolated from porcine eyes [77]. Matrigel coating on PLA nanofibrous scaffolds failed to improve the morphology, pigmentation, or barrier function of human RPE cells compared to uncoated PLA membranes; however, it prevents RPE extracellular matrix detachment and permits the culturing of RPE cells on aged and degraded scaffolds [83].

#### 4.2.3. Laminin-Attached Nanofibrous Scaffolds for 3D Cultures of RPE Cells

Laminin has been identified as a potent promoter of RPE adhesion and functional development [69,82]. Treharne et al. developed methacrylate-based copolymers with poly(ethylene glycol) (PEG) chains to improve hydrophilicity, decrease nonspecific protein absorption, and provide a spacer arm for attaching laminin and laminin-derived peptides, such as YIGSR [84]. YIGSR peptides and laminin, added to functionalized fibers, significantly impacted ARPE-19 cell adhesion and survival. The combination of GRGDSP peptide with YIGSR-modified nanofibers enhanced cellular monolayer formation. A laminin-coated PLLA membrane supports RPE cell growth and development, resulting in a robust monolayer before transplantation [82]. Porous laminin-coated 70 nm PLLA nanofibrous scaffold enhanced RPE cell proliferation and functional monolayer formation [82]. When implanted into the subretinal space of rats, no adverse immune response occurred at week 4 without immunosuppressants. Abbasi and O’Neill reported advancement in surface engineering through a novel priming technique using Poly-L-ornithine (PLO) before laminin coating [78]. PLO pretreatment enhanced laminin layer adsorption and uniformity on hydrophobic PLLA nanofibers. The surface exhibited improved hydrophilicity and supported superior proliferation, barrier function, and phagocytic activity of human embryonic stem cell-derived RPE cells. RPE cells express multiple integrin subunits, crucial for transplant growth and survival [85]. RPE cells formed organized hexagonal or polygonal monolayers on α5β1 integrin-binding peptides with 12 amino acid-coated PCL nanofiber scaffolds [74]. The integrin-binding peptide-coated PCL nanofibrous scaffold improved RPE cell attachment, proliferation, and viability. However, limbal epithelial cells on fibronectin-coated PDLLA nanofibrous scaffold did not resemble epithelial cells, showing higher expression of mesenchymal/fibroblast markers [86].

#### 4.2.4. 3D Cultures of RPE Cells on Nanofibrous Scaffolds for Retinal Transplantation

RPE cell transplantation is a promising strategy for replacing damaged RPE [87]. Subretinal injection of RPE cell suspension is insufficient, as cells fail to organize into a functional monolayer without a supportive substrate [88]. This requires a scaffold, a synthetic BM, to support transplantation, guide polarization, and promote epithelial layer formation. Subretinal transplantation models in rabbits and rats have been developed [73,82]. An advanced nanofibrous scaffold, including PLLA and SF blends, demonstrated excellent biocompatibility with minimal inflammatory response, even without systemic immunosuppressants. While RPE cells remain viable on PCL and PCL-collagen nanofiber scaffolds, maintaining their complete functional phenotype is challenging. Zimmermann et al. observed declining *RPE65* expression over time [77]. Future work should focus on developing conditions that sustain RPE functions long-term. Integrating RPE cells with other retinal cell types on 3D scaffolds is emerging for comprehensive vision restoration. RPE cells attached to ultrathin PDLLA nanofibrous scaffolds formed epithelial-like monolayers [89,90]. After one month, the RPE identity was confirmed by RPE65 and ZO-1 markers, making it suitable for in vivo transplantation [89]. The RPE-laden scaffolds showed good incorporation into the host retina over 8 weeks [90]. PLCL nanofibrous scaffolds, modified with plasma processing, enabled the formation of functional human embryonic stem cell-derived (hESC)-RPE monolayers [91]. Plasma treatment increased the hydrophilicity of hydrophobic electrospun PLCL fibers. hESC-RPE monolayers on collagen IV-coated and plasma-treated PLCL membranes showed RPE-specific markers expression, pigmentation, polarity, and uniform ZO-1 expression. Comparative transcriptome analysis of human induced pluripotent stem cell-derived RPE (iRPE) cells on scaffolds evaluates key marker genes and identifies batch-to-batch variability. Phelan et al. showed that iRPE cell maturation on electrospun soy protein nanofibrous scaffolds reduces variance in transcriptional profiles, suggesting more consistent cell products [92].

### 4.3. 3D Culture of Other Ocular Epithelial Cells on Nanofibrous Scaffolds

The avascular and transparent structure of the cornea provides refractive power, while the conjunctiva offers lubrication and immune defense. The cornea is a transparent tissue consisting of 5–7 layers of stratified squamous epithelial cells. The conjunctiva is mucous tissue extending from the corneal limbus to the inner eyelids, composed of stratified squamous nonkeratinized epithelium and loose stroma. Goblet cells are type I and specialized for mucin secretion. Cell types II–V are squamous stratified non-goblet cells and represent 85–90% of conjunctival epithelial cells. Epithelial defects in the cornea and conjunctiva may be caused by chemical, radiation, burn, or infectious injuries. Recent goals of ocular surface tissue engineering are creating a functional, stratified epithelium on a biocompatible carrier. Current therapeutic strategies for severe ocular surface damage are limited.

#### 4.3.1. 3D Culture of Corneal Epithelial Cells on Nanofibrous Scaffolds

Rabbit corneal epithelial cells on PLA nanofibrous scaffolds, dispersed by outstretching lamellipodia, firmly adhere to nanofibers with high porosities and large surface areas [93]. The cells exhibit polygonal shapes and interconnect, potentially forming an intact epithelium during extended culture. Surface wettability modification of PLA scaffolds with cellulose nanofibrils increases epithelial cell proliferation. PCL and plasma-treated PCL nanofibrous scaffolds are biocompatible for corneal and limbal epithelial cell survival and proliferation [94]. Plasma-treated PCL nanofibers showed better cell adhesion and proliferation, with cells closely attached through tight junctions and distinct borders. In contrast, cells on PCL nanofibrous scaffolds were randomly distributed with large intercellular spaces. Better adhesion on plasma-treated PCL nanofibrous scaffolds may be due to increased hydrophilicity. Poly(glycerol sebacate) (PGS) and chitosan blended with PCL enhanced hydrophilicity, creating scaffolds mimicking native corneal properties [95]. However, no significant differences in metabolic activity or cellular organization were observed in the blends. Polyvinyl acetate and type I collagen blends increased tensile strength from 0.1 MPa to 3.5 MPa, matching native corneal tissue (3–5 MPa) [96]. Aligned polyvinyl acetate-collagen scaffolds showed superior mechanical strength and light transmittance versus random counterparts. Stromal keratocytes responded to fiber alignment, but for epithelial engineering, alignment requirements remain unclear. Human corneal epithelial cells did not show alignment-guided growth like keratocytes, indicating different responses to topographical cues. On aligned PLLA nanofibrous scaffolds, keratocytes adopted elongated morphology along fiber direction, mimicking native stromal lamellae [97]. Yan et al. showed that corneal epithelial cell proliferation was favored on random gelatin-PLLA nanofibrous scaffolds [98], while keratocytes showed higher proliferation on aligned scaffolds.

#### 4.3.2. 3D Culture of Conjunctival Epithelial Cells on Nanofibrous Scaffolds

Different electrospun scaffolds, including collagen, PVA, PAA, and PCL, are used for culturing conjunctival tissue explants, with varying effectiveness. PCL and PVA are poor substrates for goblet cell expansion compared to biopolymer hydrogels and silk films [99]. PAA nanofibrous scaffolds support limited proliferation of human conjunctival goblet cells for 2 weeks. Conjunctival epithelial cells proliferate well on PGS/PCL and SF/PLCL nanofibrous scaffolds [100,101]. SF/PLCL scaffolds support stratified epithelium with *MUC5AC*-positive goblet cells for tear film protection. These scaffolds show high immune compatibility without adverse reactions or pro-inflammatory cytokine upregulation. After 3-day culture on PLA nanofiber scaffolds, conjunctival epithelial cells adhere firmly with polygonal shapes [93,102]. Compared to unmodified scaffolds, PLA nanofibrous scaffolds coated with cellulose nanofibrils and silk peptide improve cell proliferation [102]. Surface modifications influence cell viability and differentiation, with transplanted PLA nanofibrous scaffolds showing ocular biocompatibility and induced multilayered epithelium with secretory goblet cells. PLA scaffolds with silver nanoparticles demonstrate broad-spectrum antimicrobial activity against bacteria and fungi [93]. These functionalized scaffolds prevent postoperative infections (significant causes of graft failure and morbidity) and facilitate sustained, localized drug delivery. This approach overcomes the limitations of traditional eye drop treatments, which require frequent administration and are characterized by poor bioavailability.

#### 4.3.3. 3D Culture of Limbal Epithelial Cells on Nanofibrous Scaffolds

Limbal epithelial stem cells are responsible for the continuous renewal of the corneal epithelium. Ultrathin (4 μm) transparent PDLLA nanofibrous scaffolds have been developed for limbal epithelial cell cultivation [86]. Fibronectin-coated PDLLA nanofibrous scaffolds demonstrated epithelial cell expansion and cultivation. Notably, unlike the regular cobblestone epithelial morphology on fibrin gel, cells on the PDLLA nanofibrous scaffold showed a shift toward a mesenchymal, pro-fibroblastic phenotype. Additionally, fibronectin coating maintained limbal stem cell markers. The primary challenges remain in ensuring stable maintenance of the correct epithelial phenotype. Thus, evaluation is needed to determine if laminin coating maintains limbal epithelial cells in an epithelial phenotype rather than mesenchymal transition.

### 4.4. 3D Culture of Esophageal, Intestinal, and Colon Epithelial Cells on Nanofibrous Scaffolds

The human intestine is a complex organ fundamental to nutrient absorption, drug metabolism, and immune barrier function. The gut epithelium comprises several differentiated cell types, each performing a specialized function. The distribution of these cell types differs between the small and large bowel [103]. Enterocytes are the most prominent cell type in the intestinal epithelium, responsible for absorbing nutrients and water. The secretory cell types include goblet cells that secrete mucins, enteroendocrine cells that secrete hormones, and Paneth cells that release antimicrobial factors to protect stem cells. In vitro research has relied on 2D cell culture models, primarily Caco-2 cells grown on microporous inserts. Caco-2 cells are human colorectal adenocarcinoma cells that differentiate into a polarized epithelium with small intestine enterocyte characteristics upon reaching confluence [104]. A key limitation of 2D models is the formation of epithelial monolayers with supra-physiological barrier tightness. Additionally, 2D models can induce non-native cellular phenotypes, like P-glycoprotein overexpression, which affects pharmacological assessments [105]. The emergence of 3D culture models using nanofibrous scaffolds represents a significant advancement in addressing these limitations.

#### 4.4.1. 3D Cultures of Intestinal and Colon Epithelial Cells on Nanofibrous Scaffolds

Nanofibrous scaffolds are superior substrates as they structurally mimic the topography and porosity of the intestinal BM. The passive adsorption of ECM proteins (e.g., type I collagen) onto hydrophobic polymer surfaces, such as PET, improved surface wettability and promoted the robust adhesion and proliferation of Caco-2 cells [105]. Caco-2 cells cultured on PET nanofibers form a “leakier,” more physiological barrier (TEER ≈ 220 Ω·cm^2^) compared to the hyper-tight barriers of 2D cultures (>500 Ω·cm^2^). This resulted in a markedly improved correlation for predicting the transport of paracellular markers, such as Lucifer Yellow, and drugs like atenolol. Wang et al. functionalized SiO_2_ nanofibers with deoxycholic acid (DCA), a bile acid known to be implicated in cancer progression, to introduce biochemical cues relevant to the colorectal cancer microenvironment [106,107]. This study compellingly demonstrated that the biochemical microenvironment is as vital as physical topography because both normal (HIEC) and cancerous (HCT116 and SW480) intestinal cells cultured on DCA-modified nanofibrous scaffolds exhibited significantly increased resistance to radiotherapy and cisplatin chemotherapy. While functional outcomes are often observed, the underlying molecular mechanisms and signaling pathways remain fully characterized. Su et al. report an electrospinning method that replaces the conventional metallic collector with a patterned, conductive polymer, poly(3,4-ethylenedioxythiophene)-poly(styrenesulfonate) (PEDOT: PSS), integrated within a microfluidic device [108]. Thus, nanofibrous scaffolds are generated directly within the microfluidic chip, thereby eliminating the requirement of delicate transfer procedures that risk structural damage. Caco-2 cells cultured on an aligned nanofibrous scaffold showed enhanced differentiation (2.6-fold higher alkaline phosphatase activity). They formed a more robust barrier, characterized by a higher TEER, compared to cells on randomly oriented nanofibers. This study established that physical fiber alignment is a potent cellular signal. Thus, applying a nanofibrous scaffold to primary cells or organoids would be a valuable next step.

A significant challenge in intestinal 3D culture is the coexistence of multiple epithelial cell types. 3D culture of multiple intestinal cell types is achieved through systems like intestinal organoids. These models are created from stem cells or isolated crypts and differentiate into various cell types, such as enterocytes, goblet cells, and Paneth cells. Recently, human intestinal organoids, which contain differentiated epithelial cell types, have been successfully generated when using a nanofiber matrix as a differentiation substrate [109]. Poling et al. demonstrated that piezoelectric polyvinylidene fluoride-trifluoroethylene (PVDF-TrFE) scaffolds are fully biocompatible with human intestinal organoid development from human pluripotent stem cells, yielding organoids with normal histology and transcriptomic profiles [109]. Notably, the scaffold environment accelerated morphogenesis, with intestinal spheroids forming several days earlier than in standard culture, suggesting that the scaffold provides beneficial developmental cues. Duodenal and colonic epithelium models containing various intestinal cell types were constructed by culturing isolated mouse tissues from duodenal and colonic crypts on patterned electrospun PLA nanofibrous membranes with crypt-like topography [110]. Li et al. modified the patterned nanofibrous membranes using Matrigel to enhance the biocompatibility [110]. When these in vitro intestinal epithelium models were utilized to test probiotic adhesion abilities, the adhesion rates of probiotics on the colon epithelium models were consistently higher than those on the duodenal epithelium models.

PMMA-PVP nanofibrous scaffolds were produced by adding noncharged water-soluble PVP to increase wettability and control the hydrophilicity of PMMA nanofibers [111]. A confluent cell monolayer was established after ~8–10 days of Caco-2 cell culture on the PMMA membrane. After 10 days, the cells had become differentiated columnar enterocytes, characterized by microvilli structure on the apical side and expression of tight junction proteins. The permeability profiles of dextran and metformin, obtained using a microfluidic system, confirm the Caco-2 monolayer’s ability on a nanofibrous scaffold to demonstrate transport models of macromolecules and small molecules across intestinal epithelium. Intact Caco-2 cell monolayers formed on PLA nanofibrous scaffolds and developed a barrier to small molecules on PLA nanofibrous scaffolds after 4 days of culture [112]. Caco-2 cell monolayers formed microvilli and tight junctions, showing higher differentiation properties than those on the polycarbonate microporous membrane in the traditional Trans-well.

#### 4.4.2. 3D Culture of Esophageal Epithelial Cells on Nanofibrous Scaffolds

Various synthetic polymers have been utilized in esophageal reconstruction [113]. It is essential to find conditions supporting the adequate growth of epithelial cells on scaffolds. Kuppan et al. produced aligned nanofibrous scaffolds of PCL, PCL-gelatin, Poly(3-hydroxybutyrate-co-3-hydroxyvalerate) (PHBV), and PHBV-gelatin [114,115,116]. Esophageal epithelial cells adhered in cobblestone morphology and proliferated on PCL and PCL-gelatin nanofibrous scaffolds, with PCL-gelatin showing significantly higher cell proliferation [114]. Human esophageal epithelial cells showed better proliferation in the PHBV nanofibrous scaffold than in the PHBV-gelatin scaffold [114]. When epithelial and smooth muscle cells were seeded, both cell types attached and proliferated, forming two distinct layers that mimic in vivo anatomy. Higher collagen type IV expression occurred under coculture conditions than in individual epithelial cells after 7 days, suggesting that muscle cells enhance BM protein production. Laminin expression was higher in independently grown epithelial cells on PCL and PCL-gelatin scaffolds compared to cocultured cells. The nanofibrous topography provides an environment for epithelial cell differentiation. Jensen et al. showed that esophageal epithelium cells could be cultured on PLGA and PCL/PLGA nanofibrous scaffolds in a hollow organ bioreactor [117]. After 14 days, scaffolds supported epithelial, smooth muscle, and glial cell phenotypes. Nylon 6 (N6)/SF nanofibrous mats were produced with chitosan and collagen layers to enhance antimicrobial properties [118]. The N6/SF scaffold showed better epithelial cell viability than the N6 scaffold, with increased viability correlating to more bilayers. By contrast, the layer-by-layer mats showed more potent inhibition of *S. aureus* than *E. coli* and improved healing of esophageal defects in vivo.

### 4.5. 3D Culture of Kidney Epithelial Cells on Nanofibrous Scaffolds

Kidney epithelial cells form the lining of kidney nephrons, the functional units that filter blood and produce urine. A bio-artificial kidney aims to replicate renal function by combining kidney cells with engineered biomaterial scaffolds. However, kidney tissue engineering remains challenging owing to the high complexity of mature kidneys [119]. Key challenges in renal tissue engineering using nanofibrous scaffolds include achieving the specialized, polarized phenotype of renal epithelial cells, ensuring formation of a confluent monolayer with functional transporters, and promoting cell infiltration into the scaffold to create a 3D, viable tissue construct [120]. Nanofibrous scaffolds provide a biomimetic microenvironment that replicates the fibrillar architecture of the native kidney BM [121]. Kidney epithelial cells can be obtained from various sources, including primary human proximal tubule epithelial cells (PTECs), immortalized human kidney (HK)-2 cells, human kidney RC-124 cell lines, and Madin–Darby Canine Kidney (MDCK) cells. MDCK cells are widely used as a model for studying epithelial cell behavior due to their characteristics, such as apicolateral and cell junctions [122].

#### 4.5.1. Effects of Physical and Chemical Properties of Nanofibrous Scaffolds on 3D Cultures of Kidney Epithelial Cells

The physical and chemical properties of the nanofibrous scaffold directly dictate the attachment, morphology, proliferation, and specialized function of kidney epithelial cells. On smaller PCL nanofibers (~1.1 µm), human kidney RC-124 cells showed lower viability [112]. It is theorized that at this critical size, cells can neither fully wrap around a single fiber nor effectively span across multiple fibers, leading to suboptimal attachment. Larger fibers provide a more favorable substrate for anchoring and spreading [123]. Thicker PCL nanofibers (e.g., 550 nm vs. 200 nm) provide more effective topographical cues for contact guidance and directing the morphology of MDBK cells [26]. Cells cultured on random nanofibers exhibit a traditional cobblestone epithelial morphology. In comparison, aligned nanofibers are a powerful tool for directing kidney epithelial cells to adopt an elongated, organized morphology, mimicking the structure of native renal tubules. Both Madin-Darby Bovine Kidney (MDBK) and RC-124 cells cultured on aligned fibers abandon their typical cobblestone shape, elongating and aligning parallel to the fiber direction, accompanied by reorganization of the actin cytoskeleton into aligned stress fibers. This effect is robust and overrides cues from the underlying substrate chemistry [26,123]. Standard electrospun scaffolds are often dense, limiting cell penetration to the top layers. Cross-sectional confocal imaging reveals that cell migration is limited to the top surface (~10–20 µm) of standard scaffolds. In contrast, cryogenic PCL nanofibrous scaffolds, with their significantly larger pores, permit cells to migrate deep into the structure, as visualized by imaging up to 200 µm into the scaffold, forming a genuine 3D cellular construct [123].

#### 4.5.2. 3D Culture of Kidney Epithelial Cells on Functionalized Scaffolds

While a fibrous topography benefits kidney epithelial cell growth, bioactive signals are essential for maintaining functional renal epithelial phenotype over long culture periods. Simple, synthetic modifications may replace complex and expensive natural protein coatings. Sobreiro-Almeida et al. produced blends of PCL and decellularized kidney ECM by electrospinning for culturing kidney tubule cells [124]. They observed that metabolic activity, proliferation, and protein contents in HK-2 cells increased with ECM concentrations in PCL scaffolds. ZO-1 was expressed in cells cultured on ECM-containing nanofibrous scaffolds but not on PCL scaffolds. Laminin is vital for the kidney BM and cell attachment [125]. Inclusion of laminin increased Young’s modulus of the PCL nanofibrous scaffold [42]. RC-124 kidney cells attached to PCL and laminin-blended nanofibers up to 14 days and penetrated the scaffold, with cells becoming more elongated in the laminin-blended group [42].

Nanofibrous scaffolds of poly(3-hydroxybutyrate) (PHB) showed better mechanical properties, while salt-leached scaffolds had higher wettability, porosity, and air permeability [126]. Monkey epithelial kidney cells (Vero cells) attached better to nanofibrous mats than salt-leached scaffolds, but showed low adhesion to PCL nanofibrous scaffolds [127]. PTECs cultured on nonbioactive scaffolds lose monolayer integrity over time [128,129]. However, coating PCL nanofibrous scaffolds with L-DOPA and collagen IV enabled the formation of a complete PTEC monolayer [130]. Polymers functionalized with hydrogen-bonding units (ureido-pyrimidinone or bis-urea) allowed noncovalent incorporation of bioactive molecules into the polymer matrix [131,132]. Bioactive PCL nanofibrous scaffolds with ECM peptides using ureido-pyrimidinone (UPy)-modified peptides maintained tight monolayers and epithelial function under perfusion culture [129,131]. Van Gaal et al. found that UPy-modified L-DOPA incorporated into UPy-polymer films and PCL scaffolds failed to improve PTEC monolayers, even with collagen IV [132]. However, L-DOPA with collagen IV induced tight monolayers on PCL-UPy nanofibrous scaffolds. Screening showed that a coating of bisurea-conjugated L-DOPA, collagen IV, and laminin effectively induced renal epithelial monolayers, while individual ECM-mimicking peptides did not. A synthetic bisurea-catechol additive replicated the effects of the complex coating, producing quality monolayers with preserved epithelial markers and function [133].

### 4.6. 3D Culture of Skin Epithelial Cells on Nanofibrous Scaffolds

The skin is susceptible to injuries, from acute burns to chronic ulcers, which pose significant clinical challenges. Traditional wound care often fails to facilitate tissue repair. Skin tissue engineering has emerged as a promising field, using biomimetic scaffolds to guide cellular processes and promote functional tissue regeneration. With fiber diameters in nanometers, nanofibrous scaffolds closely resemble native dermal collagen’s fibrillar structure [134]. This topographical similarity, combined with high porosity and a large surface-area-to-volume ratio, creates an ideal environment for skin cells to attach, proliferate, and migrate, recapitulating initial stages of tissue development and repair. Keratinocytes are specialized epithelial cells and the most abundant type in the epidermis [135]. Although HaCaT keratinocytes are an immortalized cell line, they conserved markers of keratinocyte differentiation, including markers of terminal differentiation [136]. In this review, we analyze the roles of nanofibrous scaffolds, such as fiber diameter and topography, in modulating keratinocyte behavior.

#### 4.6.1. 3D Culture of Keratinocytes on Nanofibrous Scaffolds

Normal human epidermal keratinocytes (NHEK) from neonatal or adult skin are the most utilized keratinocytes in primary cell culture. Keratinocytes were sensitive to fiber diameter, appearing smaller and more spherical on thinner nanofibers. When immortalized human keratinocytes (NCTC2544) and skin fibroblasts (149BR) were cultured on randomly oriented PVA nanofibrous scaffolds, keratinocyte proliferation peaked on nanofibers with 305 nm diameter, similar to native ECM collagen fibrils [137]. Fibroblast proliferation was reduced on thin nanofibers (≤161 nm) but comparable to the control on thicker nanofibers. These results indicate that nanofiber diameter is critical for tissue engineering scaffolds, as cells respond to changes in this topographical feature. Adding SF to hydrophobic PCL improves surface wettability, correlating with enhanced proliferation and viability of primary human epidermal keratinocytes [138]. SF incorporation into PCL enhances mechanical properties by providing stiffness and intermolecular interactions. Layer-by-layer constructs, combining a mechanically robust layer with a bioactive one, show promise as advanced skin substitutes. In addition, dissolving microneedle matrix polymer provides an advanced example of therapeutic delivery. Hyaluronic acid microneedles loaded with salvianolic acid B achieve scar treatment with improved long-term stability, highlighting how bioactive microneedle scaffolds extend the potential of skin repair strategies [139].

Nanofibers made of tilapia skin type I collagen showed good bioactivity with HaCaTs, though fish collagen has low mechanical strength and lacks antibacterial activity [140]. Zhou et al. demonstrated that biomimetic electrospun nanofibers from tilapia fish collagen and bioactive glass (BG) promoted the adhesion, proliferation, and migration-related gene expression of HaCaT cells [141]. The tilapia-derived collagen provides a biomimetic base, while BG nanoparticles release inorganic ions (Ca, P, Si) for antibacterial effect and stimulate new blood vessel formation. Baran et al. produced PLLA shell and EGF-encapsulated collagen core nanofibrous scaffolds with a bilayer structure by gelling GeIMA between nanofibrous membranes to imitate skin layers [142]. EGF in nanofibers increased the proliferation of HaCaT cells and 3T3 fibroblasts, while cocultured bilayer membranes formed interlocked polygonal keratinocyte cells. HaCaT cells and fibroblasts showed good attachment on both sides of a bilayer *β*-glucan ester electrospun membrane with upper hydrophobic and lower hydrophilic layers [143]. Poly(acrylonitrile-co-methyl acrylate) (P(AN-MA)) nanofibers gain bioactivity by adsorbing proteins from culture serum, enabling keratinocyte adhesion and proliferation [144]. Skin fibroblasts infiltrated the scaffold, and keratinocytes cultured on fibroblasts in P(AN-MA) nanofibrous mats formed a stratified epidermal-like structure, advancing engineered tissue development.

The surface topography of a scaffold can guide keratinocyte behavior. The rough PCL/type I collagen nanofibrous scaffold did not stimulate immortalized human keratinocyte migration [145]. However, coating a rough electrospun nanofibrous scaffold with thin collagen gel created a smoother, multiscale surface that increased cell motility. This promigratory effect actively triggers an integrin β1-mediated signaling cascade, leading to the upregulation of cell motility regulators like Rac1 and CDC42, increased matrix metalloproteinase activity, and enhanced laminin-332 deposition, promoting persistent migration. The electrospun collagen nanofibrous scaffold showed low cell adhesion in NHEK and normal human oral keratinocytes (NHOK) compared to polystyrene surfaces [146]. Type I collagen and laminin promoted adhesion of proliferating NHEK and NHOK compared to collagen nanofibers alone, while fibronectin-coated nanofibers showed similar adhesion to uncoated ones. HaCaT cells showed stable proliferation on nanoporous anodic aluminum oxide membranes with collagen nanofibers, but at lower rates compared to those on APTES-modified alumina pores [147]. SF nanofibrous scaffolds supported excellent adhesion of NHOKs and fibroblasts, showing enhanced performance compared to SF films due to higher surface area and porosity [148]. Type I collagen promoted NHOK adhesion on SF nanofibrous scaffolds, while laminin- and fibronectin-coatings showed lower adhesion activity. Noh et al. demonstrated enhanced NHOK and fibroblast adhesion on chitin nanofibrous scaffolds coated with type I collagen [149]. Chitin and SF hybrid scaffolds were produced by simultaneous electrospinning in opposite directions [150]. The hydrophilicity improved by combining with chitin, and both blend and hybrid scaffolds supported NHEK attachment, with blend scaffolds showing greater effectiveness.

Mussel adhesive protein (MAP) incorporation increased adhesive properties and biocompatibility of PGLA/PCL nanofibrous scaffolds [151]. In vitro experiments show that MAP-incorporated scaffolds promote adhesion, proliferation, and migration of HaCaTs and human fibroblasts. In HaCaT and human fibroblast coculture within the scaffold, HaCaTs express keratinocyte differentiation markers CK10 and CK14, while fibroblasts secrete collagen and fibronectin. Borges-Vilches et al. developed PCL/Gelatin electrospun nanofibrous scaffolds with *Pinus radiata* bark extracts, showing antioxidant and anti-inflammatory properties for wound healing [152]. The extract-loaded nanofibrous scaffold enhanced HaCaT cell growth, attachment, and proliferation, promoting cell migration toward the scratch area in the wound healing assay. Bacakova et al. attributed the beneficial effect of plasma treatment on cell adhesion to new oxidized structures on the membrane surface, increased surface wettability, and surface stiffness [153]. Plasma treatment enhanced HaCaT adhesion and growth on PLA nanofibrous scaffolds [153]. Keratinocytes adhered and grew preferentially on scaffolds of lower fiber densities, likely due to larger void spaces between nanofibers.

#### 4.6.2. Nanofibrous Scaffolds in Skin Regeneration

Nanofibrous scaffolds are explored for applications such as wound healing and skin regeneration [154,155]. When applied to full-thickness wounds in rats, electrospun collagen nanofibrous mats demonstrated a significant acceleration of early-stage healing compared to standard gauze dressings. Histological analysis revealed a more prominent proliferation of capillaries and fibroblasts in the wounds treated with the nanofibrous matrix, indicating enhanced neovascularization and dermal regeneration [146]. Fish collagen/BG scaffolds led to faster wound closure, more complete re-epithelialization, greater collagen deposition, and more robust angiogenesis [141]. In another skin defect mouse model, on day 7 after dressing of bilayer *β*-glucan ester electrospun membrane onto the wound, epithelialization was evident in the membrane-treated group, and most of the wound area was covered with a continuous epidermis [143].

### 4.7. 3D Culture of Salivary Gland Epithelial Cells on Nanofibrous Scaffolds

The parotid, submandibular, and sublingual glands, as well as numerous minor glands, secrete saliva. A functional artificial salivary gland can be engineered to treat xerostomia (dry mouth), a condition caused by irreversible damage to saliva-secreting epithelial cells, such as those resulting from radiation therapy and Sjögren’s syndrome [156].

#### Culture of Submandibular Ductal Salivary Gland Cells on Nanofibrous Scaffolds

Salivary epithelial cells grown on nanofibers showed reduced focal adhesion proteins, mimicking mature salivary tissue. By contrast, cells on flat surfaces formed extensive focal adhesions, which are artifacts of 2D culture [157]. The downregulation of focal adhesion proteins on nanofibers led to reduced cell spreading and self-organization into 3D-like clusters [157]. PLGA scaffolds supported growth and branching morphogenesis of embryonic submandibular salivary gland cultures. A curved, concave geometry on nanofiber scaffolds mimics acinar structure and signals polarization [158]. Higher curvature increased occludin expression and apical localization in SIMS and Par-C10 cells, and increased aquaporin-5 expression in Par-C10 cells. Cantara et al. showed that bioactive molecules independently regulate cell proliferation and apicobasal polarity [44]. Laminin-111 promoted apical occludin localization without affecting proliferation, while chitosan enhanced proliferation but disrupted polarity. Elastin was incorporated into PLGA nanofibers via blending and surface conjugation [159]. The elastin-blended scaffold was more compliant, while the covalent scaffold was stiffer. Both promoted apicobasal polarity, but the elastin-blended scaffold better promoted cell clustering and contact. PGS was incorporated into PLGA scaffolds using core-shell electrospinning to enhance compliance [160]. PGS/PLGA nanofibrous scaffolds promoted cell penetration and tight junction protein localization. Coculture with NIH3T3 fibroblasts facilitated epithelial tissue reorganization. Electrochemical cell impedance spectroscopy (ECIS) measures TEER and enables real-time assessment of epithelial monolayers on nanofiber scaffolds [161]. Using ECIS-TEER, barrier formation by SIMS cells on PLGA nanofibers was tracked, demonstrating its utility for evaluating scaffold designs.

## 5. Basic Research and Clinical Applications of Nanofiber Scaffolds

Nanofibrous scaffolds are applied in basic research for various tissues by mimicking the natural ECM to study cell behavior and tissue regeneration. Different polymers can create scaffolds with tailored mechanical and chemical properties, allowing for specific research applications. From the basic research, nanofibers have clinical applications in cancer research, tissue engineering, and regenerative medicine for multiple diseases. Nanofibers can be loaded with various drugs and released in a controlled way to a specific tumor site, improving efficacy and reducing systemic toxicity [162]. The versatility of nanofibrous drug delivery enables the targeted delivery of a variety of drugs. Among various nanofiber fabrication techniques, electrospinning is the most studied and appears to offer the most promising results for tissue engineering [163]. Besides the cells and growth-controlling bioactives, nanofibrous scaffolds play the central role in tissue engineering and regenerative medicine, which is analogous to the role performed by the ECM in vivo [164]. Nanofibers are scaffolds for vascular tissue engineering, neural tissue engineering, musculoskeletal tissue engineering (including bone, cartilage, ligament, and skeletal muscle), and skin tissue engineering. However, nanofibrous scaffolds need optimization of their composition and structure for in vivo tissue regeneration. Electrospun nanofiber composites with hydrogels can overcome the limitations of both for soft tissue regeneration.

## 6. Future Outlook

Despite significant advancements in current nanofiber production for epithelial cell culture, several challenges and future directions exist. Natural polymers often exhibit poor mechanical strength and batch-to-batch variation, while synthetic polymers may produce toxic degradation byproducts or lack inherent bioactivity [31]. Tailoring the balance between mechanical properties and biological performance is an ongoing challenge for biomedical applications of nanofibrous scaffolds. While electrospinning is effective at the lab scale, scaling up production for industrial applications remains a hurdle. Advancements in equipment and process speed are required. Future studies should aim to combine nanofibers with other advanced strategies to mimic better the natural ECM, such as integrating them with hydrogels, which provide a hydrated, three-dimensional environment that mimics natural tissue. The current models often lack a functional vasculature, which is critical for nutrient supply and for studying systemic responses. The stable and long-term incorporation of a diverse immune cell population is also tricky, limiting the scope of immunological studies. Finally, the use of patient-derived induced pluripotent stem cells (iPSCs) or primary cells to populate these scaffolds will pave the way for creating personalized disease models. This will enable the testing of therapeutic strategies tailored to an individual’s genetic background, a key goal of precision medicine. Polymer scaffold and drug-conjugated nanocarrier systems can be developed not only in model tumor biology but also in advanced targeted cancer therapy [165].

## 7. Conclusions

This review provides insights into the function of nanofibrous scaffolds in the fabrication of 3D culture systems for various types of epithelial cells. Suitable nanofibrous scaffolds in 3D culture of various epithelial cells of different tissues are shown in Table 3. The convergence of nanofibrous scaffold technology and advanced 3D cell culture methodologies has produced a new class of in vitro epithelium models with unprecedented physiological relevance. The central strategy is to develop biomimetic scaffolds that replicate the natural ECM and BM in the epithelium, thereby providing the necessary signals to guide epithelial cells to organize, polarize, and differentiate correctly. By mimicking the native ECM architecture, these platforms successfully guide various epithelial cells to form complex, functional, and differentiated tissues that were previously unattainable in a lab setting. Thus, the optimal nanofibrous scaffold for epithelial cell regeneration will likely require an integrated design that combines nano-topography, macro-geometry (curvature), biochemical functionalization (laminin and elastin), and tuned mechanical properties (compliance). Future investigations should be focused on synthesizing these composite scaffolds and utilizing functional assays, such as TEER, to accelerate their development and optimization.

## Figures and Tables

**Table 1:** Advantages and disadvantages of natural, synthetic, and hybrid polymer scaffolds.

Polymer Type	Examples	Advantages	Disadvantages
Natural	Collagen, Gelatin, Chitosan, Silk Fibroin	- Inherently bioactive (e.g., containing RGD cell-binding sequences) - Excellent biocompatibility - Biodegradable	- Poor mechanical strength - Potential for immunogenic response - Significant batch-to-batch variability.
Synthetic	PLLA, PCL, PLGA	- Highly tunable mechanical properties - Predictable and controllable degradation rates - High purity - Consistency	- Inherently hydrophobic, which can hinder initial cell attachment - Lack of native bioactive sites, often requiring surface functionalization - Acidic degradation byproducts can induce local inflammation.
Hybrid	Blends (e.g., PCL-Gelatin)	- A combination of the mechanical strength and stability of synthetic polymers with the inherent bioactivity of natural polymers creates a superior composite material.	- Fabrication can be complex. - Potential for delamination between material phases - Achieving a homogenous blend can be challenging.

**Table 2:** Advantages and disadvantages of primary and immortalized bronchial and lung cells in 3D culture on nanofibrous scaffolds.

Cell Source	Description and Examples	Advantages	Disadvantages
Primary cells	- Harvested directly from donor tissue and used with minimal passaging. - NHBE cells - HSAE cells	- High physiological relevance: capable of forming a fully differentiated and pseudostratified mucociliary epithelium. - Gold standard: considered the most accurate representation of in vivo tissue. - Patient-specific models: enable personalized medicine approaches using cells from patients with specific diseases (e.g., cystic fibrosis).	- Limited lifespan: Finite proliferative capacity restricts the scale and duration of experiments. - Donor-to-donor variability: Significant biological differences between donors can affect reproducibility. - High cost and limited availability: more expensive and difficult to procure than cell lines.
Immortalized cell lines	- Cells that have been genetically modified to proliferate indefinitely in culture. - BEAS-2B: SV40-transformed bronchial epithelial cells. - Calu-3: adenocarcinoma-derived, forms tight junctions and secretes mucus. - A549: alveolar adenocarcinoma-derived.	- High reproducibility: genetically homogenous, providing consistent results across experiments. - Ease of culture and scalability: robust and easy to expand to large numbers for high-throughput screening. - Cost-effective: readily available and inexpensive to maintain.	- Altered phenotype: Genetic modifications can lead to non-physiological behavior. - Incomplete differentiation: often fails to differentiate into a complex and pseudostratified epithelium fully. - Tumorigenic origin: Many lines are derived from cancers, which may not reflect normal cell biology.

**Table 3:** Summary of epithelial cell culture on nanofibrous scaffolds.

Tissue Type	Cell Type	Nanofiber Composites	Specific Purposes	Ref.
Bronchial and Lung epithelium	NHBE cells	PCL	Formation of differentiated pseudostratified Epithelium on multilayer scaffolds	[50]
		PCL	Tracheal frame using 3D printing	[51]
	Primary human alveolar epithelial cell (pneumocyte)	Polyurethane	Coculture of endothelial cells Lung-on-a-chip model	[64]
	Porcine tracheobronchial epithelial (PTBE) cell	PCL/Chitosan	Air-liquid interface culture	[166]
	Bronchial epithelial cells (16HBE)	PCL, PCL/CA, PCL/CAP, PCL/EC	Culture of various types of epithelial cells	[167]
	Bronchial epithelial cell (MLE-12)	PVA	Laminin-coated and peptide-blended scaffold	[43]
		PVA	Coculture of fibroblasts	[55]
		PCL	*S. aureus* infection model	[66]
	Bronchial epithelial cell (Calu-3)	PCL/Chitosan	All-*trans* Retinoic Acid-loaded	[65]
	Lung epithelial cell (A549)	Gelatin	Air–liquid interface, microfluidic	[52]
		Polyurethane	Aligned and non-aligned nanofibers	[54]
		PVA/Silk sericin	Epithelial-mesenchymal transition induced on scaffolds	[56]
		PVA/Collagen	Epithelial-mesenchymal transition induced on scaffolds	[57]
		PGLA	Coculture of human fetal lung fibroblasts	[61]
		PCL/Collagen	Nanofiber on a microfluidic chip	[168]
		PCL/Gelatin	Coculture of endothelial cells	[169]
		PDMS/PMMA	Combined with microfluidics	[170]
	Lung epithelial cell (NCI H441)	PCL	Coculture of endothelial and immune cells	[62]
Retinal pigment epithelium	Primary RPE cell	SF/PCL/Gelatin	Similar thickness to native Bruch’s membranes	[73]
		PLGA/Collagen	Formation of sheet-like microvilli	[75]
		PET/PLCL	Fiber diameter-dependent adhesion	[76]
		PCL/Collagen	Stable long-term culture on scaffolds	[77]
		Polyamide	A colony-like distribution of polygonal cells	[79]
		PDLLA	Compared to the polyester membrane	[80]
		PLLA	Functional RPE monolayer on laminin-coated scaffolds	[82]
		PLA	Matrigel-coated scaffolds	[83]
		PDLLA	Ultrathin scaffold with frame	[89,90]
		PCL	Surface modification by plasma surface treatment	[171]
		Gelatin/Chitosan	Appropriate adhesion of cells on the substrate	[172]
	human RPE cell (ARPE-19)	PCL	Integrin-binding peptides-coated	[74]
		PAN	FA-treated nanofiber	[81]
		PEG/methacrylate	Peptide and laminin-attached	[84]
	Stem cell-derived RPE cells	PLLA	Laminin-coated scaffolds	[78]
		PLCL	Plasma processing, Collagen IV coating	[91]
		Soy protein/PCL	Blow electrospun soy scaffolds	[92]
Other ocular epithelium	Human corneal epithelial cells	PCL	Modified by helium-oxygen (He/O_2_) plasma discharge	[94]
		PCL/PGS, PCL/chitosan	Random and aligned scaffolds	[95]
		Polyvinyl acetate/ collagen	Random and aligned scaffolds	[96]
		Gelatin/PLLA	Random and aligned scaffolds	[98]
	Rabbit corneal epithelial cells	PLA	Coated by cellulose fibril and Ag nanoparticle	[93]
	Primary limbal epithelial cells	PDLLA	Induction of mesenchymal phenotype in fibronectin-coated scaffolds	[86]
		PCL	Modified by helium-oxygen (He/O_2_) plasma discharge	[94]
		PLGA	Combined pattern of nanofiber on microfabrication	[173]
	Rabbit conjunctival epithelial cells (HCjEC)	PLA	Coated by cellulose fibril and Ag nanoparticle	[93]
	Conjunctival goblet cells	Collagen/PAA/PCL, PVA	Growth of goblet cells in PAA scaffolds	[99]
	human conjunctival epithelial cells (HCjEC)	PGS/PCL	Aligned scaffold	[100]
	Rabbit conjunctival epithelial cells	SF/PLCL	Implantation of cell-seeded scaffold	[101]
		PLA	Coated by cellulose nanofibrils and/or silk peptide, transplanted in vivo	[102]
	Human corneal epithelial cells (HCE-T), Human limbal epithelial cells	PCL	Limbal epithelial cell expansion	[174]
Esophageal, intestinal and colon epithelium	Esophageal epithelial cells	PHBV/PCL	Gelatin-blended aligned scaffolds	[114,116]
		PHBV	Gelatin-blended	[115]
		PCL/PGLA	In bioreactor	[117]
	Porcine esophageal epithelial cells	PLA	Nanoporous fiber scaffold	[175]
		PLLC	Fibronectin immobilization on the scaffolds	[176]
	Human intestinal epithelial cells	PVA/SiO_2_	Modified with deoxycholic acid	[106]
		Nylon 6/silk fibroin	Chitosan and collagen-coated	[118]
	Intestinal organoid epithelial cells	PVDF-TrFE	Intestinal organoid on a nanofiber	[109]
	Colon epithelial cells (Caco-2)	PET	Collagen-coated scaffolds	[105]
		PVP	Aligned nanofiber on a microfluidic	[108]
		PLA	Modified with Matrigel, Crypt-like pattern	[110]
		PMMA-PVP	Scaffold in a microfluidic system	[111]
		PLA	Monolayer	[112]
		PCL/Cellulose	Other epithelial cell culture	[167]
Kidney epithelium	human primary tubular epithelial cells (PTEC)	PCL	UPy-Urea-modified	[128,129]
	Conditionally immortalized proximal tubule epithelial cells (ciPTEC)	PCL	Coated with l-DOPA and collagen	[130]
		PCL	Incorporation of UPy-DOPA in PCL-diUPy	[132]
	Human kidney-2 (HK-2) cells	PCL	Decellularized kidney ECM-blended	[124]
		PCL	UPy-modified, peptide-blended	[131]
		Bis-urea/PCL	Peptide additive	[133]
	human kidney epithelial cells (RC-124)	PCL	Laminin-blended	[42]
			Cryogenic electrospun random and aligned scaffolds	[123]
	Madine Darby Bovine Kidney epithelial cells (MDBK)	Chitosan/PCL	Collagen-coated, random, and aligned	[26]
	Monkey epithelial kidney cells (Vero)	PHB	Electrospinning and salt-leaching procedures	[126]
		PCL	Increased cell proliferation in thick scaffolds	[127]
		Chitosan/PCL	Hyaluronic acid scaffold layered	[177]
Skin epithelium	Primary human keratinocytes	PVA	Nanofiber diameter-dependent growth	[137]
		SF/PCL	Increased tensile strength and hydrophilicity	[138]
		P(AN-MA), Pullulan/PVA/PAA	Air-liquid interface	[144]
		Collagen	Collagen, laminin-coated	[146]
		Chitin	Collagen-coated	[149]
		Chitin/SF	Blend and hybrid scaffold	[150]
	Human keratinocytes immortalized	PCL/Collagen	Collagen-coated	[145]
	Keratinocytes (HaCat)	Tilapia collagen	Wound healing	[140]
		Collagen/bioactive glass	Wound healing	[141]
		PLLA/Collagen	Coaxial, EGF-encapsulated collagen fiber	[142]
		*β*-glucan ester	Bilayer culture	[143]
		Collagen	Anodic aluminum oxide-modified	[147]
		PLGA/PCL/MAP	Enhanced adhesive properties and biocompatibility	[151]
		PCL/gelatin	*Pinus radiata* bark extracts (PEs)-incorporated	[152]
		PLA	Plasma-treated scaffolds	[153]
Gland ductal epithelium	Salivary gland epithelial cells, SIMS and SMGC10 cell line	PLGA	Chitosan-attached Laminin-111-attached	[44]
	Ductal submandibular epithelial cell	PLGA	Elastin-attached scaffolds by blending and covalent surface conjugation	[159]
	Salivary gland ductal epithelial cells (SIMS)	PLGA	Decreased levels of the focal adhesion proteins in scaffold culture	[157]
		PLGA	Micropatterned scaffold crater	[158]
		PGS/PLGA	Coculture with fibroblasts	[160]
		PLGA	Nanofiber scaffold integrated into an ECIS-TEER Trans-well system.	[161]
		PGLA	Different solvents for the fabrication	[178]
